# Supplementary material for: Causal interplay between lactose intolerance and gut microbiota: a combined bidirectional Mendelian randomization and in vivo validation study
Source: Front Nutr. 2026 Jun 1;13:1803337. doi: 10.3389/fnut.2026.1803337 (PMC13265576; doi:10.3389/fnut.2026.1803337)
Supplement: Supplementary file 1 [file Data_Sheet_1.zip › supplementary materials/Forward/leave one out cross validation/ebi-a-GCST90027852.finngen_R12_E4_LACTONAS.pdf]

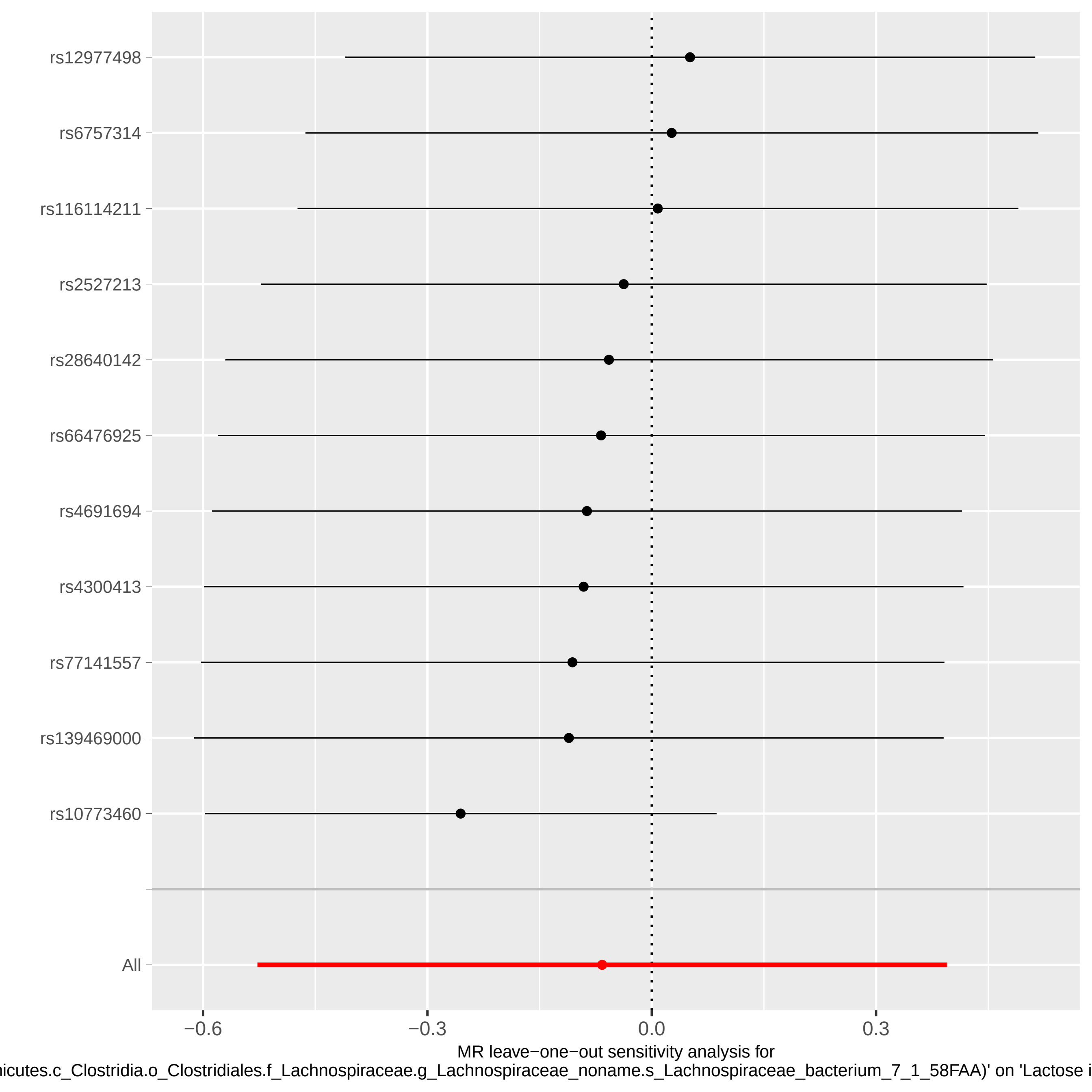
rs12977498
rs6757314
rs116114211
rs2527213
rs28640142
rs66476925
rs4691694
rs4300413
rs77141557
rs139469000
rs10773460
All
−0.6
−0.3
0.0
0.3
MR leave−one−out sensitivity analysis for
nicutes.c_Clostridia.o_Clostridiales.f_Lachnospiraceae.g_Lachnospiraceae_noname.s_Lachnospiraceae_bacterium_7_1_58FAA)' on 'Lactose i
